# Supplementary material for: Non-Thermal Plasma Jet-Treated Medium Induces Selective Cytotoxicity against Mycobacterium tuberculosis-Infected Macrophages
Source: Biomedicines. 2022 May 26;10(6):1243. doi: 10.3390/biomedicines10061243 (PMC9219627; doi:10.3390/biomedicines10061243)
Supplement: Supplementary file 1 [file biomedicines-10-01243-s001.zip › biomedicines-1686140-supplementary.pdf]

Non-Thermal Plasma Jet-Treated Medium Induces Selective Cytotoxicity against *Mycobacterium tuberculosis*-Infected Macrophages

Chae Bok Lee [1], Kang In Lee [1], Young Jae Kim [1], In Taek Jang [1], Sintayehu Kebede Gurmessa [1], Eun Ha Choi [2], Nagendra Kumar Kaushik [2] and Hwa-Jung Kim [1,*]

[1] Department of Microbiology & Medical Science, College of Medicine, Chungnam National University, Daejeon 301-747, Korea; cblee2017@cnu.ac.kr (C.B.L.); popigleton@nate.com (K.I.L.);
invivor@gmail.com (Y.J.K.); janginteak@naver.com (I.T.J.);
sintayehu.kebedeg@gmail.com (S.K.G.)

[2] Plasma Bioscience Research Center, Department of Electrical and Biological Physics, Kwangwoon University, Seoul 01897, Korea; ehchoi@kw.ac.kr (E.H.C.); kaushik.nagendra@kw.ac.kr (N.K.K.)

* Correspondence: hjukim@cnu.ac.kr; Tel.: +82-42-580-8242

Supplemental Figures

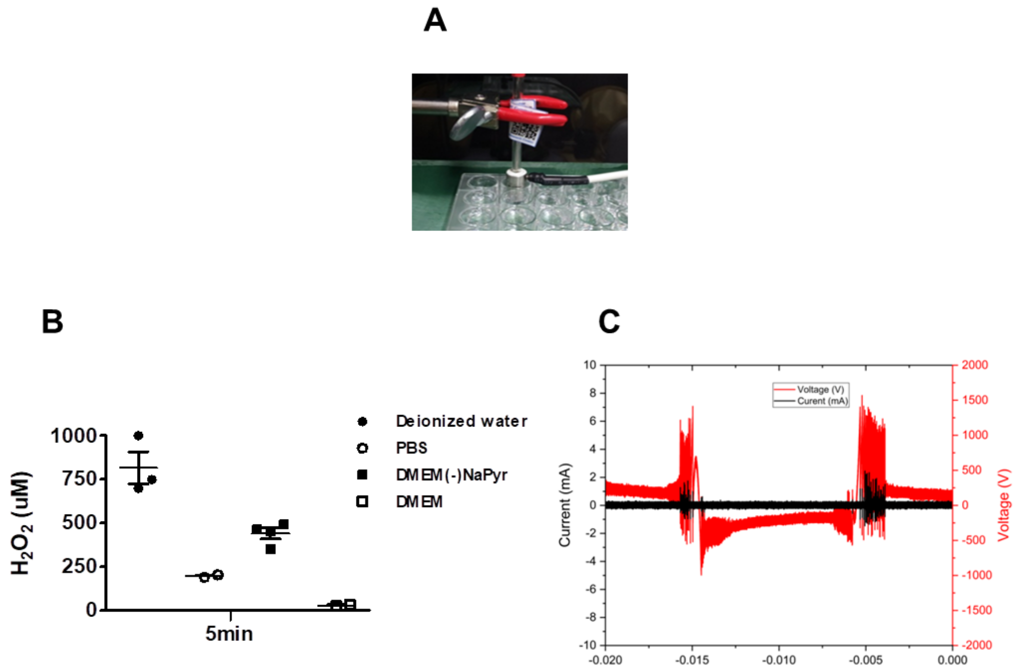

Figure S1. NTPJ source, H_2O_2 concentrations at different liquids, and electric power. (A) Photogr aph of atmospheric pressure jet source. (B) The H_2O_2 concentration in DW, PBS, DMEM without NaPyr, and complete DMEM for 5 min by NTPJ. (C) Discharge power and voltage-current wav eforms of NTPJ at 1.5 SLM (N_2 gas), V= 1.06 kV; I=4.5 mA; P =4.77 W.

A

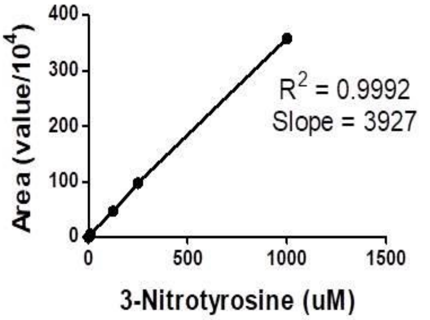

B

3-nitrotyrosine

Conc.	Time	Area	Area%
10 mM	7.796	20470100	90.828
1 mM	7.810	3589009	63.003
0.25 mM	7.811	987528.6	37.667
0.125 mM	7.811	477588.8	24.558
12.5 uM	7.851	60850.3	3.768
6.25 uM	7.840	35416.2	2.273

C

0.1mM L-tyrosine

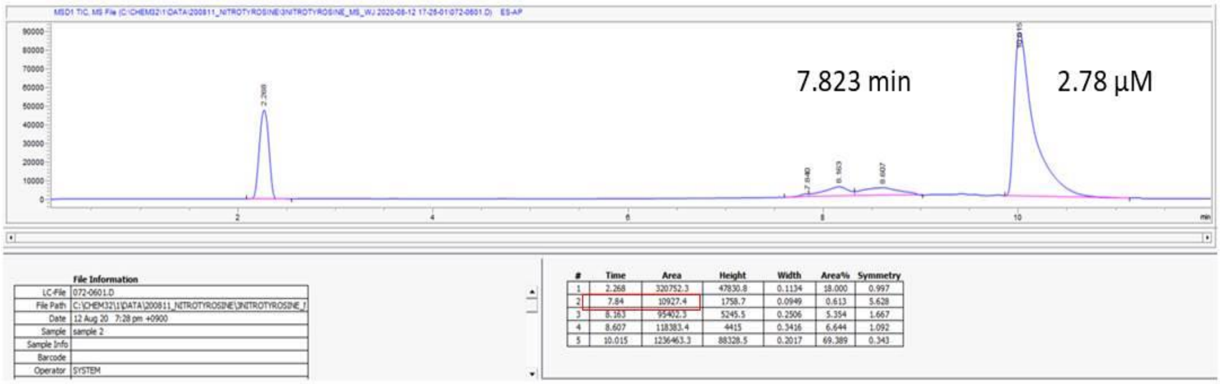

D

1mM L-tyrosine

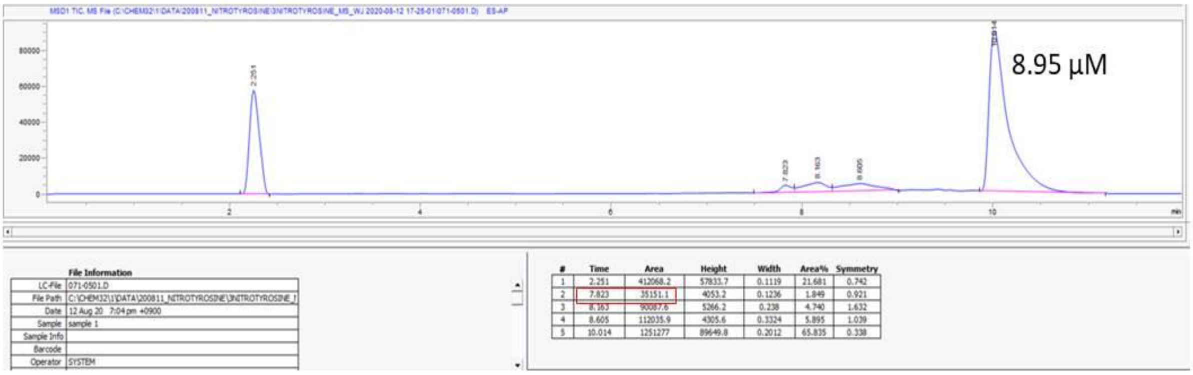

Figure S2. ONOO$^-$ quantification with LC-MS. Calibration curve of 3-NT and 3-NT generation in NTPJ treatment for 5 min. (A-B) Standard graph and area. (C-D) 3-NT value in 0.1mM or 1mM L-tyrosine induced PTM for 5 min treatment, respectively. Retention time:7.823 min ; 0.1 mM L-tyrosine, Area =10927; 1 mM L-tyrosine, Area= 35151.

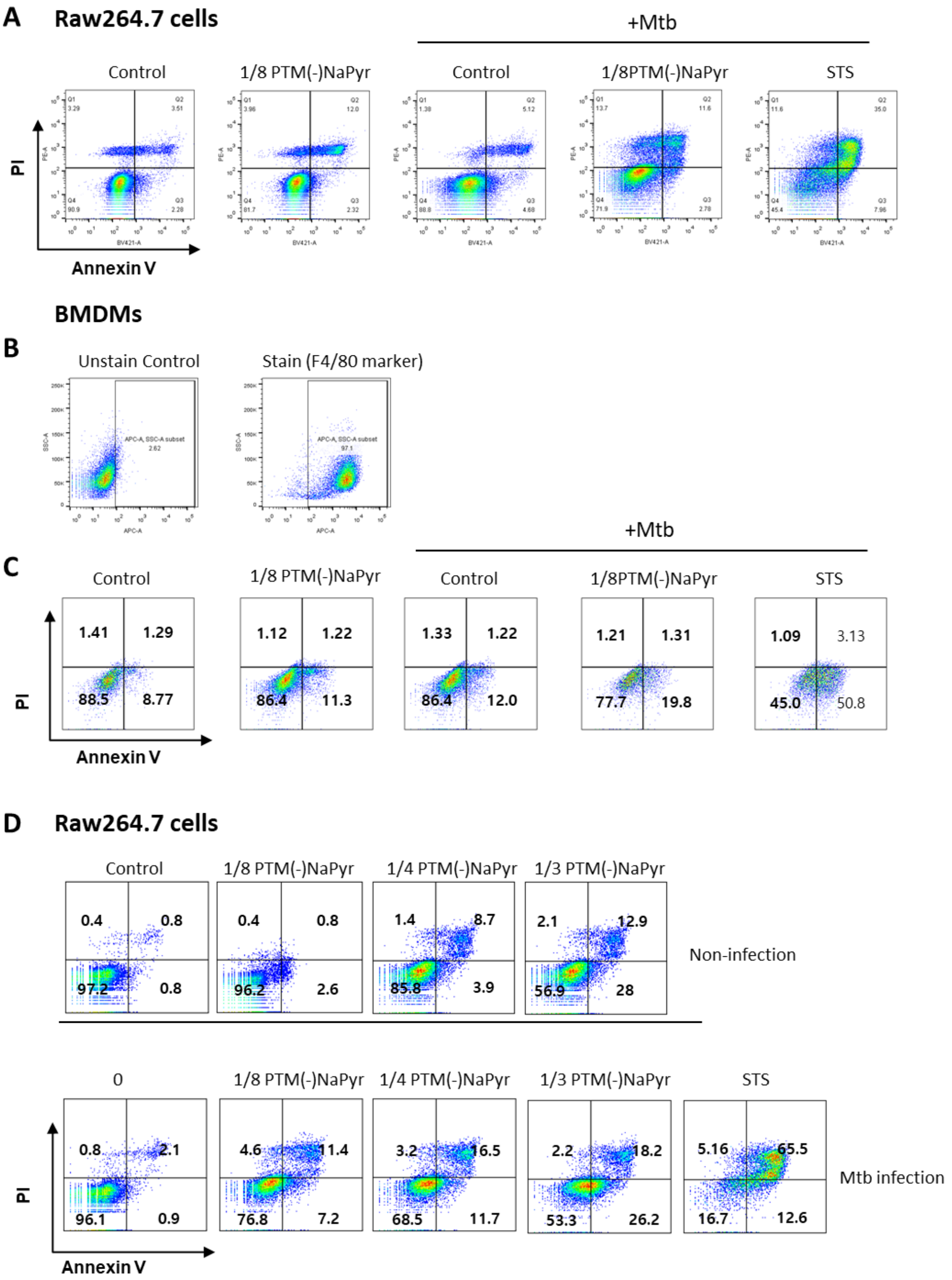

Figure S3. Cell death analysis using flow cytometry. (A) *Mtb*-infected Raw264.7 cells (B) Analysis was done by gating live-cell surface marker APC using log phase controls in BMDMs, and STS (50 nM). (C) BMDMs at MOI1 or non-infected cells were treated with 8-fold diluted PTM(-)NaPyr or STS (100 nM).

(D) Non-infected and *Mtb*-infected Raw264.7cells exposed to PTM-diluted medium at different dilution ratios (8-fold, 4-fold, and 3-fold dilution).

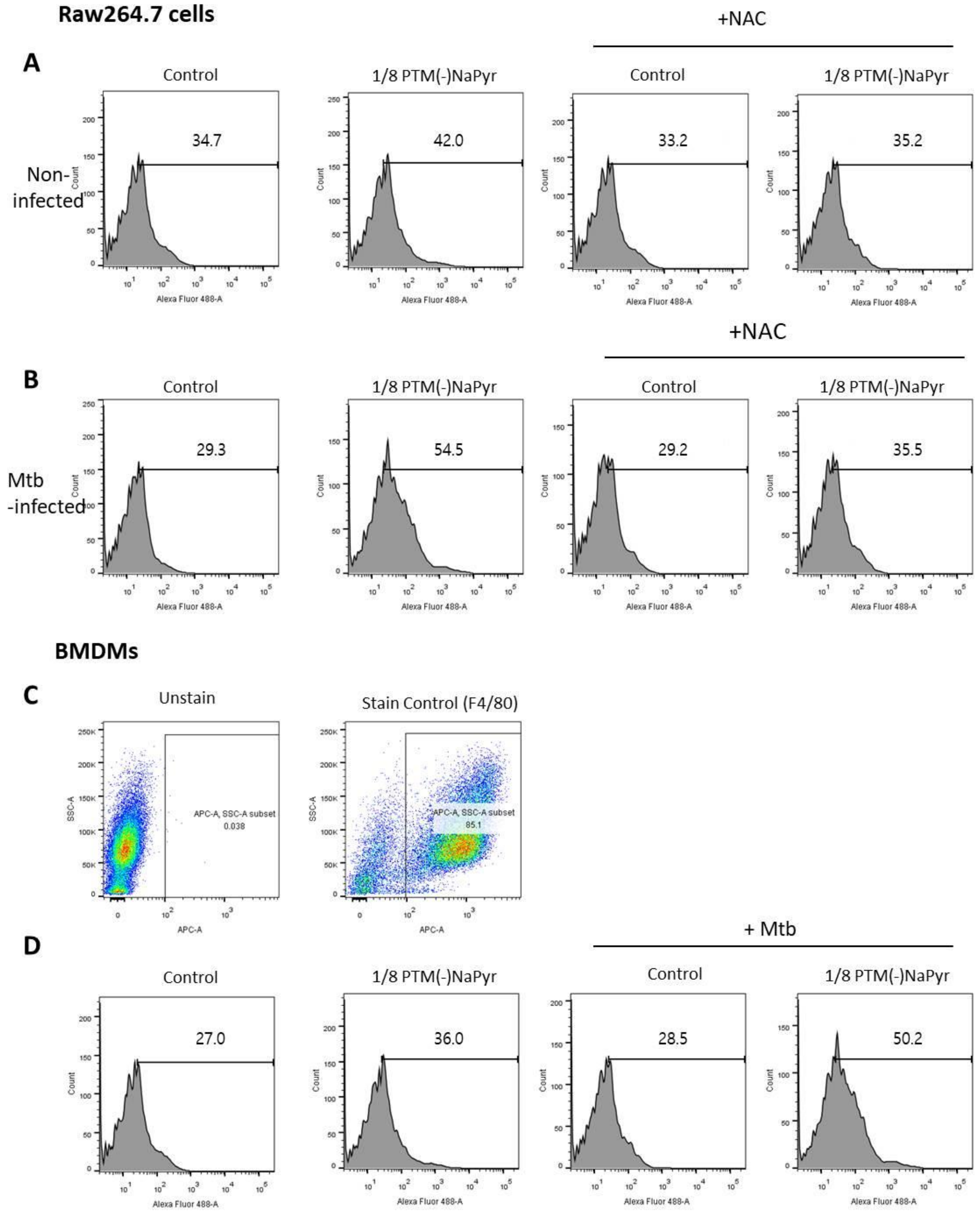

Figure S4. Intracellular ROS was measured using flow cytometry, stained with H_2DCFDA.(A-B) unninfected or *Mtb*-infected Raw 264.7 cells. (C) Analysis was done by gating live-cell surface marker APC using log phase controls in unstained BMDMs (D) Stained BMDM cells after 8-fold dilution PTM(-)NaPyr or with pre-treated NAC (10 mM).

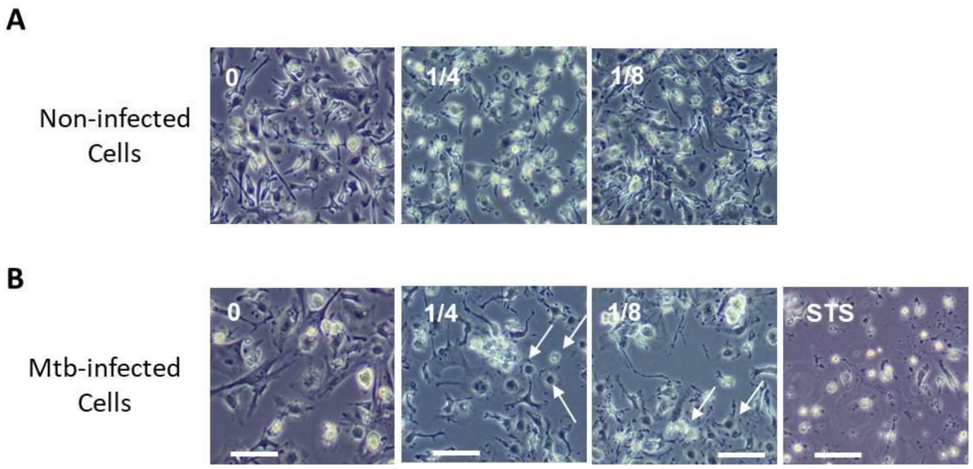

Figure S5. Images of activation or apoptosis of uninfected or *Mtb*-infected primary macrophages (BMDMs) at 24 h elapsed from 4- or 8- diluted PTM(-)NaPyr culture, respectively. BMDMs (2×105cells/12well) were infected with *Mtb* (MO1). Cells were treated with 4- or 8- PTM diluted culture, respectively. STS was used 100 nM. Like STS, 4-diluted or 8-diluted PTM culture showed morphological apoptosis similarly STS. The white arrows indicate the apoptosis of *Mtb*-infected cells. Phase contrast bright field images showing morphological changes: (A) uninfected (B) *Mtb*-infected cells. Scale bars, 10 μm.

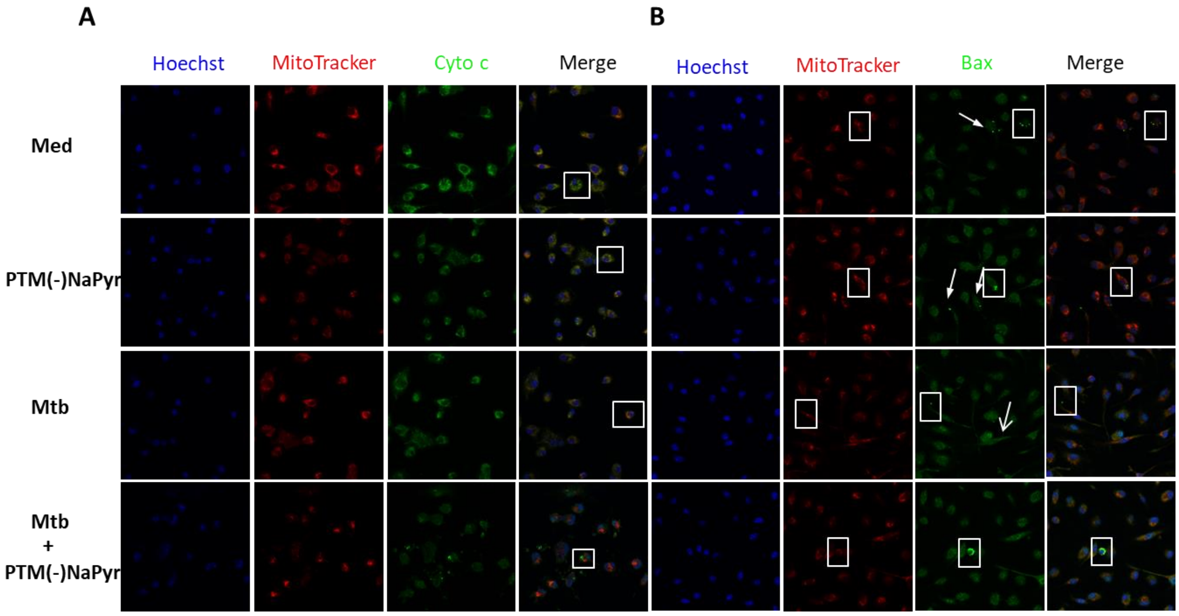

Figure S6. Changes in Cyt c and Bax localization in *Mtb*-infected cells exposed to PTM. (A-B) Confocal microscopy images showing MitoTracker (red) and Cyt c or Bax (green) staining of only medium (Med), PTM(-)NaPyr-uninfected BMDMs, *Mtb*-only infection only cells, and 8-fold diluted PTM(-)NaPyr-treated and *Mtb*-infection cells, for 18 h. (A) Cyt c, or (B) Bax. Scale bar = 10 μm.

Table S1. Oligonucleotides used to perform qRT-PCR analysis

Oligonucleotide	Oligonucleotide Sequences (5′-3′)
RT CAT F	CACTTTGGAGTTGTGCCGTG
RT CAT R	TGGCTCGGACCAGATCAGTA
RT ATM F	AGGGGAGCGAGTGTTTTGAG
RT ATM R	GCAGCAAGTTGGTAAGGCAC
RT GPx1 F	CGTGCAATCAGTTCGGACAC
RT GPx1 R	CCGAGGATAGGCCTGCATTC
RT 18S F	GTAACCCGTTGAACCCCATT
RT 18S R	CCATCCAATCGGTAGTAGCG
